# Radiological features accompanying peroneus brevis split rupture revealed on magnetic resonance imaging – a cohort study

**DOI:** 10.1186/s13047-023-00604-4

**Published:** 2023-03-02

**Authors:** Michael Huuskonen, Simon Borkmann, Alexander Bengtsson, Piotr Sobecki, Rafał Józwiak, Nektarios Solidakis, Paweł Szaro

**Affiliations:** 1 Department of Radiology, Institute of Clinical Sciences, Sahlgrenska Academy, University of Gothenburg, Gothenburg, Sweden; 2 Department of Musculoskeletal Radiology, Sahlgrenska University Hospital, Gothenburg, Sweden; 3 Applied Artificial Intelligence Laboratory, National Information Processing Institute, Warsaw, Poland; 4 Faculty of Mathematics and Information Science, Warsaw University of Technology, Warsaw, Poland; 5 Department of Descriptive and Clinical Anatomy, Medical University of Warsaw, Warsaw, Poland

**Keywords:** Tendon injuries, Magnetic resonance imaging, Ankle injuries, Tendon, Anatomy

## Abstract

**Background:**

Peroneal split tears are an underrated cause of ankle pain. While magnetic resonance imaging (MRI) is useful for diagnosis, split tears are challenging to identify. The aim of the study was to investigate the association of peroneus brevis split rupture with abnormalities of the superior peroneal retinaculum (SPR), anterior talofibular ligament (ATFL), calcaneofibular ligament (CFL), joint effusion, morphology of the malleolar groove, presence of the bone marrow oedema and prominent peroneal tuberculum.

**Methods:**

Ankle MRI cases were assessed by independent observers retrospectively in two groups: one with peroneus brevis split tears (*n* = 80) and one without (control group, *n* = 115). Two observers evaluated the soft tissue lesions, and three graded the bone lesions. Fisher’s exact test and Pearson correlation were used for analysis. The Bonferroni-Holm method (B-H) was used to adjust for multiple comparisons.

**Results:**

Only bone marrow edema in the posterior part of the lateral malleolus was significantly (*p* < 0.05) more common in the split tear group after applying B-H. SPR total rupture was seen only in the experimental group. No differences in incidence of ATFL and CFL lesions or other SPR lesions were noticed (*p* < 0.05).

**Conclusion:**

Bone marrow edema in the posterior part of the lateral malleolus is associated with peroneus split tears on MRI.

## Background

Ankle disorders are a common clinical problem. Injuries to the ankle constitute up to 10% of all visits to the emergency room (ER) [1] and about 25% [2] of all injuries to the musculoskeletal system are inversion injuries to the ankle. In turn, roughly 50% of these are sport-related [2]. Estimations based on cadaveric dissections put the incidence of peroneus brevis (PB) tendon split tears between 11% and 37% while split tears in the peroneus longus (PL) tendon are less common [3, 4]. The true incidence of split tears is unknown but most likely higher than reported in the literature due to frequent clinical misdiagnosis [5]. Radiological misdiagnosis of PB split rupture is still unknown. It is said that injuries to the peroneus tendons can also occur in conjunction with anterior talofibular ligament (ATFL), calcaneofibular ligament (CFL) or superior peroneal retinaculum (SPR), exacerbating confusion [6]. A similar inversion mechanism may cause ligament rupture and split rupture of PB. Instability following ligament injury may coexist with peroneal tendinopathy [7]. The overlapping of ligament injury and ankle instability with tendon split makes clinical diagnosis very challenging. Usually, an MRI or ultrasound examination is needed. Preoperative MRI may be vague in many of those cases. The previous study has shown that the interobserver reliability of MRI findings was relatively low [7]. Therefore, careful MRI evaluation with attention to certain features is crucial.

To our best knowledge, no previous study regarding the relationship of the PB split rupture and lateral ligament injury has been conducted before.

The pathophysiology of peroneus tendon split tears is not entirely understood [8]. It has been hypothesized that peroneus split tears arise through two primary mechanisms: chronic overuse and acute injuries [9, 10]. Injuries are more common in the PB than the PL [5, 11]. The most common location for PB tears is the malleolar groove region, while PL tears most commonly occur in the cuboid notch, which may be caused by a different mechanism [3, 4]. A traditionally described injury pattern is forced dorsiflexion causing a split of the PB, followed by the PL inserting into the split, obstructing reconnection of the halves [12]. The split spreads longitudinally if allowed to progress [5]. Split tears are the most common type of peroneal tear and complete ruptures are rare [12, 13].

Clinical examination may be inaccurate in the acute situation due to pain [9]. MRI or ultrasound can be used to obtain a comprehensive view of the ankle by surveying the tendons, but also other soft tissues and the skeleton. The modality can be applied in both acute and chronic cases [1]. While axial and oblique images are the most useful in elucidating pathology in the tendons, all three orthogonal planes should be utilized [9, 12, 14]. Despite its utility [13], MRI alone is reportedly not yet sufficient in diagnosing peroneal split tears [8]. Ankle MRI assessment in general is a demanding endeavor [1] and, furthermore, peroneal injuries are among the most challenging pathologies to identify on MRI [12]. The difficulty is in part due to the flattened appearance of the PB tendon and artifacts [13]. On images portraying PB split tears, the tendon may resemble a boomerang or cashew nut in shape as it wraps around the PL [13], but this is not always the case. The specificity of MRI in diagnosing tears in the PB and PL is about 44% and 55%, respectively, while the sensitivity is 99% and 96%, respectively [11]. False-positives and false-negatives are cited as complicating factors [3].

The International Olympics Committee (IOC) has repeatedly emphasized the importance of preventing injuries in the pathophysiology of peroneus split rupture [15]. Because of the wide spectrum of sports injuries, modifying prevention measures for each sport’s injury profile is essential [16]. Understanding the anatomical risk factors and facilitating MRI assessment would aid in both aspects, i.e., prevention and diagnostics. Previous studies regarding peroneus split rupture have been conducted on smaller sample sizes; furthermore, control groups have rarely been used, and results been mixed [17, 18]. From our understanding, no studies have been conducted to investigate the relationship between peroneus split tears, joint effusion and synovitis. Joint effusion and synovitis may associate with ankle injury and instability. These two variables are essential to the overall characteristics of the joint. They are usually the first variables a radiologist will evaluate in an MRI [19].

The aim of this study was to investigate the association of peroneus brevis split rupture with abnormalities of the superior peroneal retinaculum (SPR), anterior talofibular ligament (ATFL), calcaneofibular ligament (CFL), joint effusion, morphology of the malleolar groove, presence of the bone marrow oedema and prominent peroneal tuberculum.

## Materials and methods

### Study design

This study has a retrospective cohort design where already-existing MR images of the ankle were analyzed. The investigated parameters were compared using two groups: one consisting of patients with confirmed split tear of the PB tendon and one control group without a split tear. Additional inclusion and exclusion criteria were employed as described below. Our experimental group (Table 1) included 40 females and 40 males. The mean age was 50 ± 13 years. The right ankle was examined in 36 cases, the left one in 44 cases. The control group (Table 1) included 58 females and 57 males. The mean age was 40 ± 14 years. The right ankle was examined in 55 cases, the left one in 60 cases. Demographics of the study population included in the study are presented in Table 1. The average body mass index (BMI) was 24.1 kg/m^2^ in the experimental group and 23.7 in the control group (p > 0.05) (Table 2).

### Inclusion criteria

MRI examinations of the adult patients (age > 18 years) performed between 2018 and 2021 (*N *= 239) at the Sahlgrenska University Hospital (SU) in Gothenburg, Sweden, were eligible.

A dedicated ankle coil was used for MRI acquisition at 3.0 Tesla. Only MRI examinations with the following sequences were included: proton density (PD)-weighted with and without fat suppression, T2-weighted and T1-weighted without fat suppression.

All ankle MRI examinations included PD-weighted turbo spin echo (TSE): echo time (TE) 45 ms and repetition time (TR) 2800–5000 ms; T2-weighted (TSE): TE 60 ms and TR 3000–5000 ms; and T1-weighted: TE 11.5 ms, TR 700–750 ms. Voxel size was 0.45 × 0.53 × 3.0 mm, slice thickness 3 mm and field of view (FOV) 14 cm.

### The foot and ankle position during the examination

The patient was placed in a supine position. Ankle joint localization was maintained using a dedicated coil suited to the shape of the ankle and foot. To further lock the ankle and foot position, elastic wedge-shaped cushions were used.

### Exclusion criteria

**Fig. 1:**
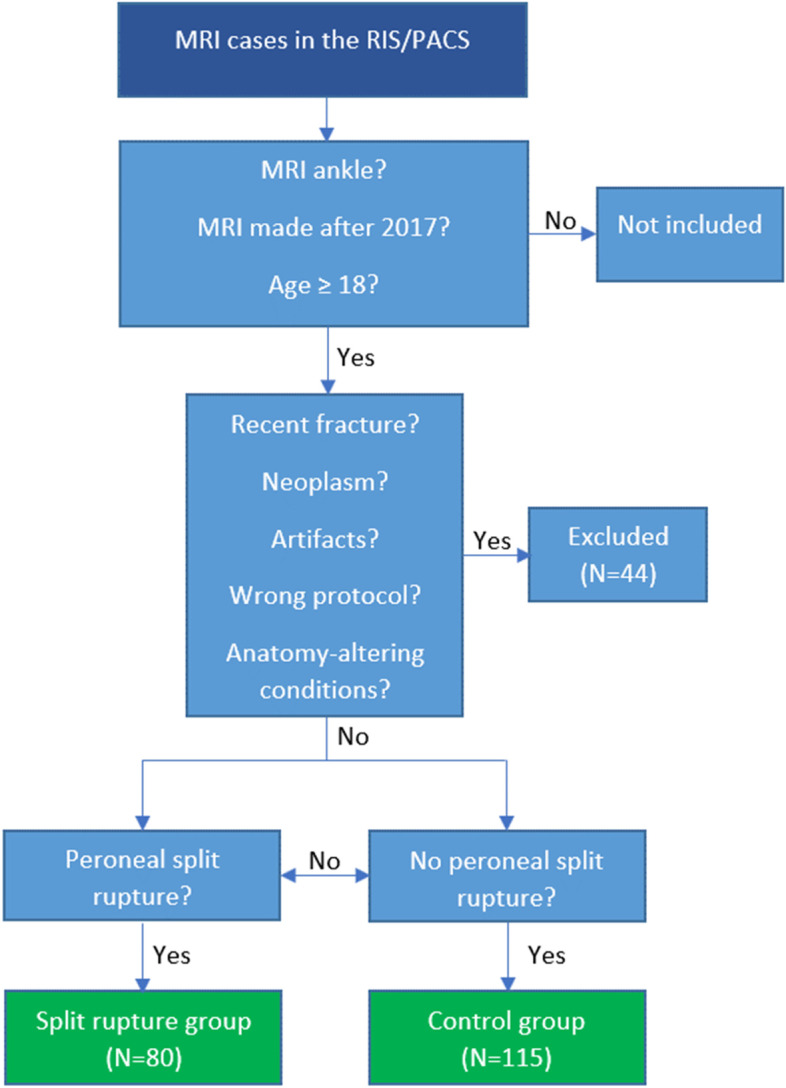
Flow chart demonstrating how magnetic resonance examinations were collected. MRI – magnetic resonance imaging, RIS/PACS – Radiological Information System/Picture Archiving and Communication System

Exclusion criteria were recent fracture (n = 2 from the control group), neoplasm (n = 2 from the control group), sequences without angled axial projections (n = 6 from the control group and 8 from the experimental group), artifacts obstructing evaluation (e.g., metal artifacts, n = 7 from the experimental group and n = 4 from the control group) and conditions which severely altered the appearance of the ankle (n = 13 from the control group and 2 from the experimental group). In total, we excluded 44 cases (17 from the experimental group and 27 from the control group), Fig. 1. These criteria were chosen to include images that allowed for proper evaluation of the peroneus tendons and minimized interfering noise from other conditions and artifacts.

### Assignment to experimental and control groups

Peroneal split tear was defined as a radiologically and clinically proved identifiable longitudinal tear of the PB tendon. All patients that met the inclusion and exclusion criteria were included in the split tear group.

The control group was assembled in a similar way. Matching exclusion criteria were applied but, in contrast, the image sequences were evaluated to *not* include any PB split tears.

### Other variables included and evaluated

The soft tissue abnormalities included in the study were abnormality in the SPR, ATFL or CFL. Abnormalities of SPR were classified as total rupture, relaxed or thickened. Abnormalities of the ATFL and CFL were assessed as one of three grades [20]: grade 1 is interstitial ligament injury which manifests on PD-weighted or fat-suppressed PD-weighted MR images as mild intraligamentous signal hyperintensity, ill-definition of the ligament and pericapsular edema; grade 2 was assessed as a focal ligament fiber; grade 3 was complete ligament fiber discontinuity [20].

The anatomical variations included in the study were the low-lying PB, os peroneum, prominent peroneal tubercle and malleolar groove shape. The presence of os peroneum was defined as a separate bone situated within the PL tendon, typically in the area inferior to the os cuboideum. If the length of the peroneal tubercle, measured from the lateral margin of the calcaneus, exceeded the width of each peroneal tendon it was defined as prominent. Three malleolar groove shapes were included: concave, flat and convex. The PB was considered low-lying if the muscle belly was seen at least at the level of the lateral malleolus at the level where the posterior talofibular ligament (PTFL) attaches. The shape was assessed at the level of where the PTFL attaches to the fibula. Using measurement tools in the Radiological Information System/Picture Archiving and Communication System (RIS/PACS), a straight line could be drawn through the groove. If the groove curved towards the center of the fibula, away from the measurement line, the groove was considered concave. If the groove neither curved nor protruded from the measurement line, it was considered flat. If the groove protruded beyond the measurement line, it was considered convex.

**Fig. 2:**
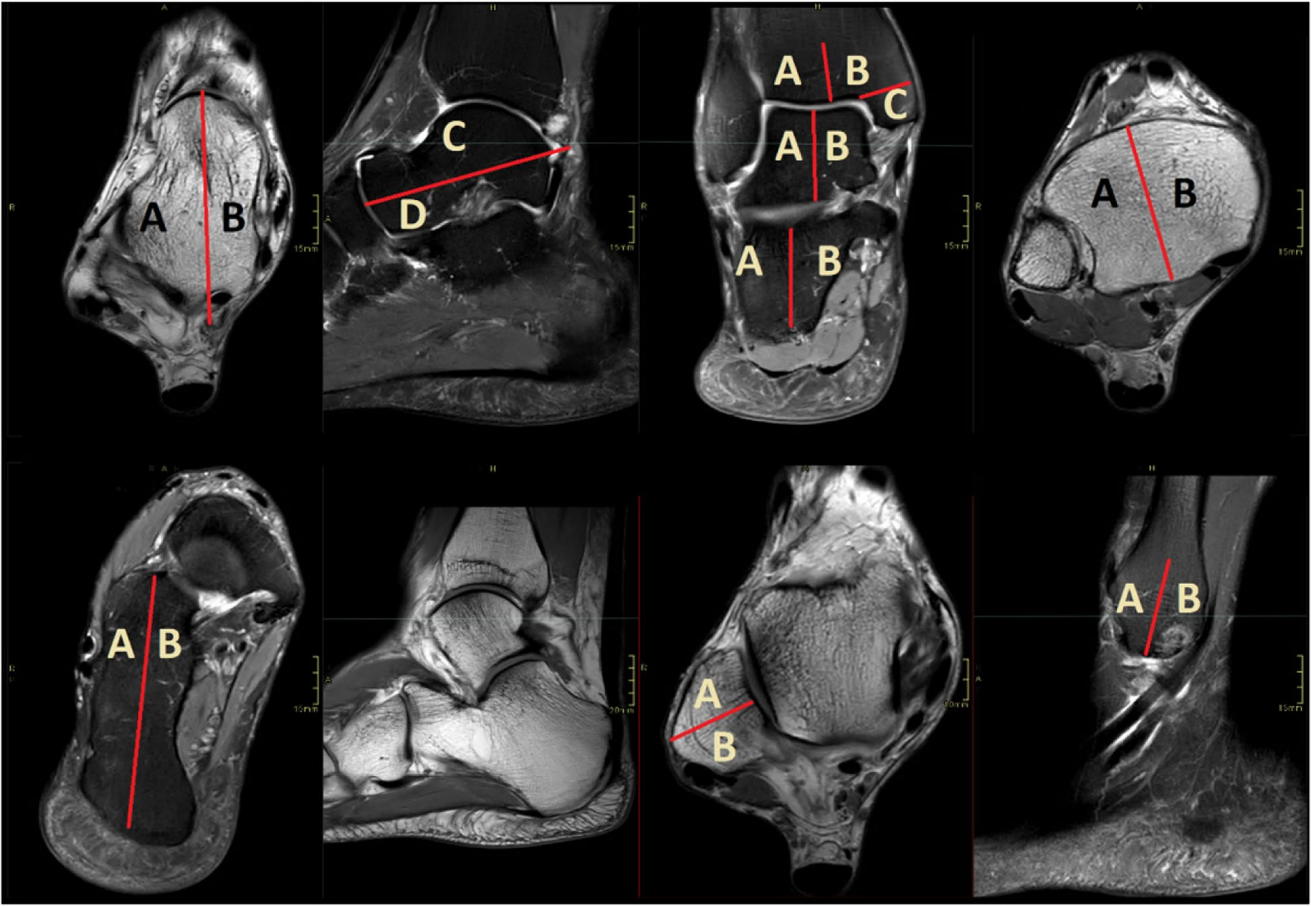
Illustrating the defined distribution areas of bone marrow edema. Bone section coded as described in Sect. 2.6. A, D, G – proton density-weighted images; B, C, E, H – T2-weighted images with fat suppression; F – T2-weighted image

To more accurately evaluate localization of bone marrow edema, the tibia, lateral malleolus, talus and calcaneus were divided into sectors (see Fig. 2). The distal tibia was divided into three parts: the medial malleolus (Tibia C), the lateral half (Tibia A) and the medial half (Tibia B). The fibula was divided into an anterior half (Fibula A) and a posterior half (Fibula B). The talus was divided both in terms of superior (Talus C)/inferior (Talus D) and medial (Talus B)/lateral (Talus A). The calcaneus was divided into a lateral half (Calcaneus A) and medial half (Calcaneus B).

No gap between the talus and the adjacent fat pad in the anterior joint recess was defined as no effusion (coded as Effusion 1). Fluid creating a gap between the talus and the adjacent fat pad in the anterior joint recess, as well as fluid expansion of the posterior recess, was defined as moderate effusion (coded as Effusion 2). If fluid also created a gap between the anterior tibia and adjacent fat pad, it was defined as severe effusion (coded as Effusion 3). Presence of the synovitis in the talocrural joint was noted.

We measured patients’ height and weight and BMI was calculated. History of trauma was considered if the patient had had an ankle injury in the last 6 months.

### Observers

All MRI examinations were evaluated using the radiology information system/ picture archiving and communication system (RIS/PACS) of the hospital (AGFA©). The data collection procedure was done separately by observers who were radiologists with 12 (PS, observer 1) and 3 years’ experience (AB, observer 2 and SB, observer 3) and a medical student (MH, observer 4) who did master’s degrees in medicine at the Department of Musculoskeletal Radiology at SU in Gothenburg, Sweden. PS and AB evaluated the soft tissue lesions. PS, SB and MH graded the bone lesions. Before scoring, the most experienced radiologist (PS) held a training session with all observers to ensure conformity in evaluation. The final decision was made by consensus.

### Statistical analysis

The proportion and percentage of feature occurrence, compared to the total number of patients in each group, was calculated (i.e., the probability of a feature occurring in each group). The analyses are based on comparisons of the probability of feature occurrence in both groups. This supposition was made due to the relatively large size of the groups. In this case, the probability was determined by analyzing feature frequency in the study groups. Binominal proportion confidence intervals (CI, 95%) were calculated using asymptotic normal approximation and Wilsons’s test, to evaluate variance. The Bonferroni-Holm method (B-H) [21] was used to adjust for multiple comparisons. The data was nominal, in sets of two unpaired groups. Hence, to determine the significance and magnitude of percentual differences, p-values and CI (95%) were calculated using two-sided Fisher’s exact test (FT). Statistical significance was always defined as p < 0.05.

Continuous variables (BMI) were found to be normally distributed. Parametric tests were used for variables with normal distribution. Differences in mean values were analyzed with Student’s t-test. A p value ≤ 0.05 was considered as statistically significant.

To investigate bivariate correlations, Pearson correlation for binominal variables was used to analyze the direction, strength (Pearson’s correlation coefficient, r) and significance of correlations.

To evaluate the inter-rater reliability of the method, the results from the two parties were compared using Cohen’s kappa coefficient for each parameter using SPSS. Cohen’s intervals were used for interpretation: no agreement (≤ 0), none to slight (0.01–0.2), fair (0.21–0.4), moderate (0.41–0.6), substantial (0.61–0.8) and almost perfect (0.81–1.0) [22]. In addition to Cohen’s kappa, cross-tabulation of percentual agreement was calculated for each parameter. Cohen’s kappa measured the degree of inter-rater reliability while the percentual agreement examined *what* differed in the evaluation.

Analyses and data visualization were performed using Microsoft Excel©, Statistical Package for the Social Sciences program (SPSS)© and Python 3.7.10 (with plotly 4.4.1, scipy 1.4.1 and statsmodels 0.10.2 libraries) in Jupyter notebook.

Ethics.

The ethical considerations of this study were primarily the management of confidential patient material and the digital storage of said data. Patients were assigned pseudo-encrypted codes through which deidentification was achieved. The Swedish Ethical Review Authority approved the study and waived the need for informed consent (number 2020-06-177 and 2021–05447).

The study was conducted in compliance with the Declaration of Helsinki. The anonymization of patient data ensured data protection following the European General Data Protection Regulation. The data were recorded in a password-protected secure database.

## Results

MRI examinations were assigned into two groups, experimental and control, following the flow chart in Fig. 1. Seventeen patients with split tears and twenty-seven patients without were excluded (to see reasons for exclusion, see exclusion criteria in Methods), Fig. 1.

### Demographics, and anthropometric and trauma history in the experimental and control groups

No statistically significant differences were found regarding demographic and anthropometric parameters were similar in both groups (p > 0.05), Tables 1 and 2. History of trauma was considerably more common in the control group (63%) than in the split tear group (40%) (p < 0.05).

**Table 1:** Main demographics of the split tear group and control group rounded to the nearest integer, including trauma parameters

Characteristic	Patients with peroneus split tears (n = 80)	Control subjects (n = 115)
Mean age ± 1 SD – years	50 ± 13	40 ± 14
Male sex – % (no.)	50 (40)	50 (58)
Female sex – % (no.)	50 (40)	50 (57)
Right side – % (no.)	45 (36)	48 (55)
Left side – % (no.)	55 (44)	52 (60)
History of trauma – % (no.)*	40 (29)	63 (64)
Mean time since trauma ± 1 SD – years	2 ± 4	2 ± 2

* As “history of trauma” could not be determined for all patients, in these calculations n = 73 for the split tear group and n = 102 for the control group.

**Table 2:** Anthropometric data

**Experimental group**			
	Weight	Height	BMI
General	75.1	1.8	23.7
Male	78.1	1.8	24.2
Female	71.9	1.7	23.2
**Control group**			
General	77.0	1.8	24.0
Male	76.0	1.8	24.2
Female	77.6	1.8	24.4

*BMI*  Body mass index

### Agreement between observers

#### Soft tissue variables

The agreement between observers in the experimental group was moderate to perfect [22]; the highest kappa was noticed in evulsion for total rupture of ATFL and the lowest for partial tear of the ATFL (Table 3). The agreement between observers in the control group was substantial [22]; the highest kappa was noticed for total rupture of SPR and the lowest for grade 1 rupture of ATFL (Table 3).

The most common findings in the experimental group were peroneal tenosynovitis (n = 27), relaxed SPR (n = 29), and thickening of SPR (n = 18; Table 3). None of the differences in the variables in the soft tissue group were statistically significant (p > 0.05; Table 3). Total rupture of SPR was seen only in the experimental group (n = 11; Table 3).

**Table 3:** Agreement between observer 1 and observer 2 in evaluation of soft tissue lesions

	Observer 1 vs. 2 (Cohen’s κ)	Experimental group	Observer 1 vs. 2 (Cohen’s κ)	Control group	p
Peroneal tenosynovitis	0.72	27	0.69	13	0.172
SPR **total rupture**	0.71	11	0.82	0	N/A
SPR **relaxed**	0.69	29	0.73	8	0.240
SPR **thickened**	0.80	18	0.66	6	0.683
ATFL **grade 1**	0.66	8	0.67	6	0.982
ATFL **grade 2**	0.59	9	0.72	6	0.413
ATFL **grade 3**	0.82	6	0.69	6	1.000
ATFL total		23		18	0.544
CFL **grade 1**	0.71	14	0.72	8	0.487
CFL **grade 2**	0.74	6	0.78	7	0.876
CFL **grade 3**	0.78	6	0.78	6	1.000
CFL total		26		21	0.757

SPR – superior peroneal retinaculum, ATFL – anterior talofibular ligament, CFL – calcaneofibular ligament, N/A – not applicable.

### Bone variables and joint fluid

In the experimental group, the Cohen’s kappa value between the most experienced observers (1 vs. 2, 1 vs. 3) was moderate to perfect, highest for bone marrow edema in the medial part of the distal tibia (without medial malleolus) and lowest for the medial part of the calcaneus, Table 4. In the control group, the Cohen’s kappa value between the most experienced observers was substantial to perfect, highest for bone marrow edema in the anterior part of the lateral malleolus and lowest for the lateral part of the distal tibia (Table 4).

In the split tear group, the Cohen’s kappa value between the most experienced observers was the lowest for “convex malleolar groove” (moderate) and the highest for concave (substantial) (Table 4). In the control group, agreement between the most experienced observers was substantial, lowest for flat malleolar groove and highest for convex.

Agreement in evaluation of os peroneum and prominent peroneal tubercule between the most experienced observers was substantial in the experimental group and moderate in the control group (Table 4).

**Table 4:** Agreement between observers and differences between groups in evaluation of bone and joint fluid. Two-sided Fisher’s exact test was used to evaluated differences. The Bonferroni-Holm method was used to adjust for multiple comparisons if Fisher test was p < 0.05

	p	Experimental group (Cohen’s κ)			n	Control group (Cohen’s κ)			n
Feature –		Observer 1 vs. 4	Observer 1 vs. 3	Observer 3 vs. 4		Observer 1 vs. 4	Observer 1 vs. 3	Observer 3 vs. 4	
Anterior part of lateral malleolus	FT p > 0.05	0.42	0.65	0.53	6	0.35	0.82	0.46	4
Posterior part of lateral malleolus	FT p < 0.001; B-H p < 0.001	0.50	0.72	0.45	19	0.22	0.81	0.49	3
Lateral part of the talar body	FT p < 0.05; B-H p = 0.264	0.56	0.78	0.67	19	0.26	0.81	0.39	12
Medial part of talar body	FT p > 0.05	0.43	0.61	0.60	11	0.47	0.73	0.61	22
Superior part of the talar body	FT p > 0.05	0.45	0.58	0.51	18	0.47	0.67	0.57	20
Inferior part of the talar body	FT p > 0.05	0.65	0.70	0.72	18	0.48	0.64	0.55	18
Lateral part of the distal tibia	FT p > 0.05	0.39	0.81	0.74	10	0.41	0.61	0.53	5
Medial part of the distal tibia (without medial malleolus)	FT p > 0.05	0.32	0.82	0.60	8	0.32	0.73	0.49	8
Medial malleolus	FT p > 0.05	0.66	0.79	0.68	13	0.42	0.70	0.54	11
Lateral part of the calcaneus	FT p > 0.05	0.39	0.58	0.65	9	0.25	0.67	0.55	6
Medial part of calcaneus	FT p > 0.05	0.42	0.51	0.60	6	0.36	0.68	0.45	7
*Normal amount of joint fluid*	FT p < 0.001; B-H p = 0.099	0.52	0.82	0.59	37	0.36	0.78	0.49	77
*Moderate effusion*	Fisher test p < 0.05; B-H p = 0.186	0.41	0.81	0.59	33	0.46	0.76	0.68	26
Severe effusion	FT p > 0.05	0.49	0.91	0.64	10	0.54	0.77	0.70	12
*Concave groove*	FT p < 0.05, B-H p = 0.132	0.39	0.65	0.59	34	0.44	0.56	0.49	26
Flat groove	FT > 0.05	0.19	0.56	0.49	24	0.25	0.47	0.39	42
Convex groove	FT p > 0.05	0.32	0.55	0.43	23	0.35	0.59	0.40	47
Os peroneum	FT > 0.05	0.41	0.78	0.63	11	0.35	0.68	0.51	8
Prominent peroneal tubercle	FT > 0.05	0.37	0.80	0.54	2	0.45	0.62	0.54	5

FT- Fisher test, H-B - Holm–Bonferroni method.

**Fig. 3:**
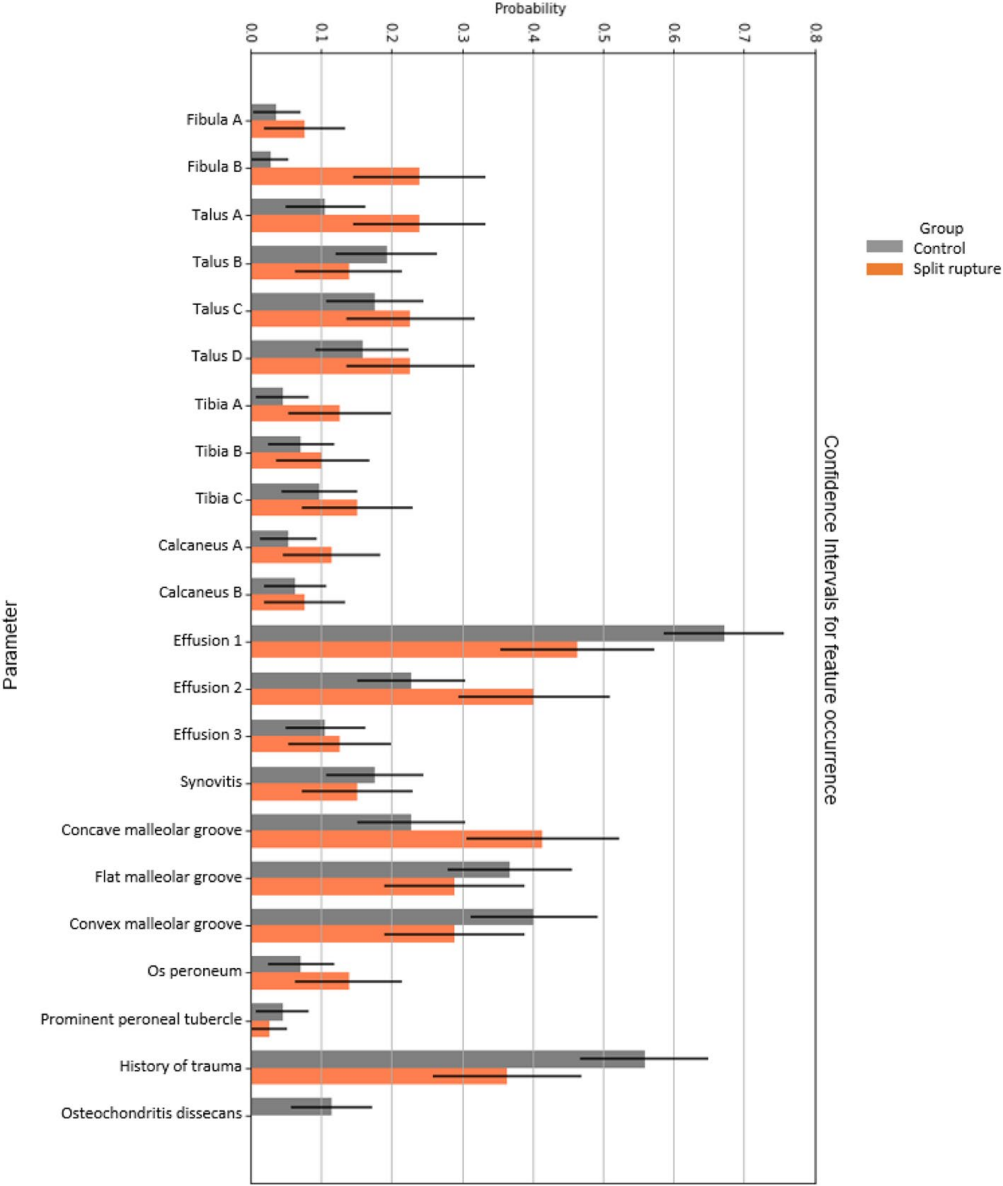
Bar graph visualizing feature occurrence in both groups. Coding of bone sections and joint fluid as described in Sect. 2.6. Error bars denote 95% confidence intervals

Most of bone variables are somewhat similar in occurrence in both groups, Fig. 3 Table 4. Group CI calculated with asymptotic normal approximation and Wilson’s test were equivalent.

Using FT statistically significant differences were identified in occurrence of presence of bone marrow edema in the posterior half of the lateral malleolus, normal and moderate increased amount of joint fluid (Table 4). After using correction for multiple comparisons only significant differences in the presence of bone marrow edema in the posterior half of the lateral malleolus was shown (Table 4).

**Fig. 4:**
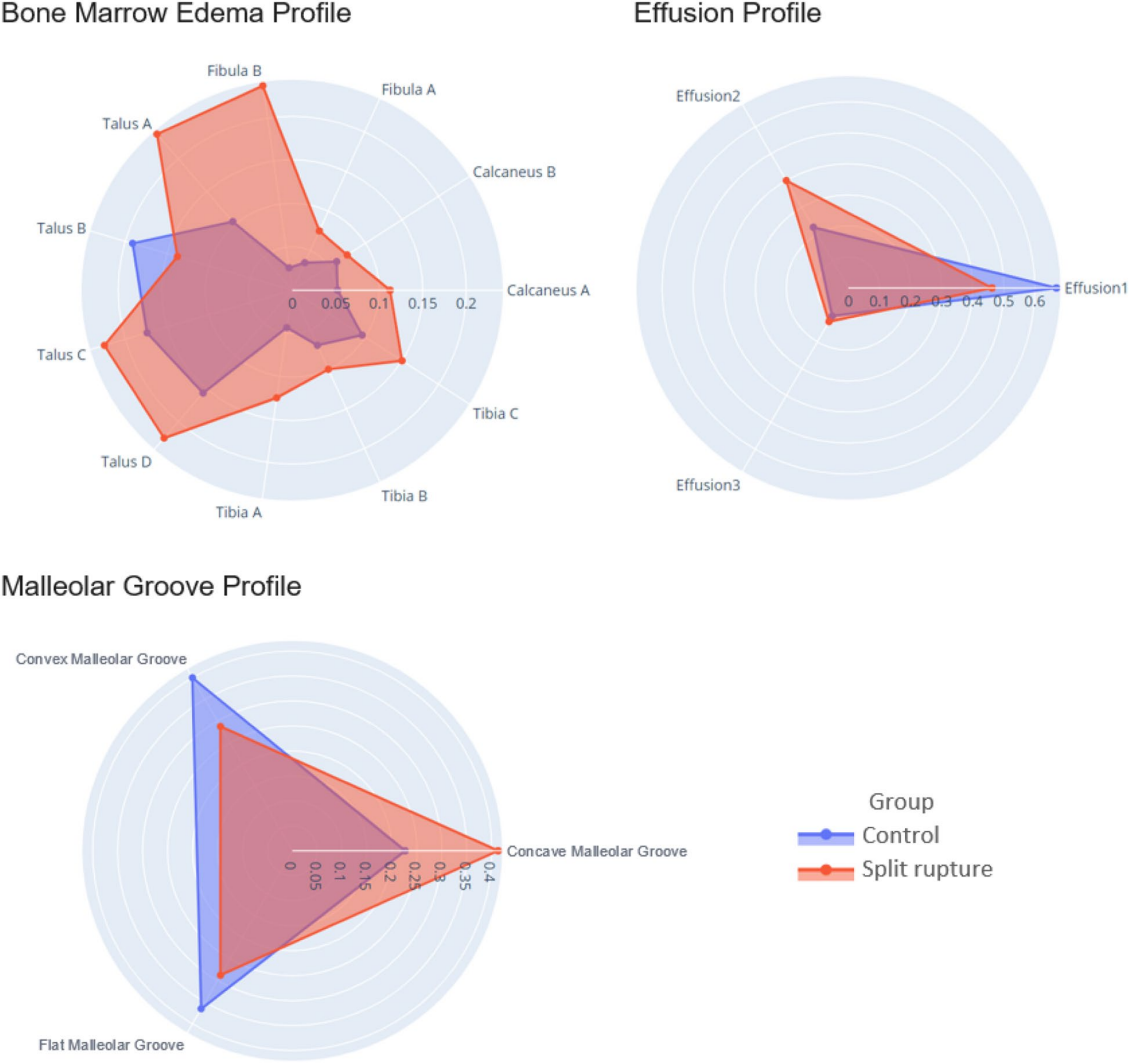
Radial plots visualizing the occurrence of bone marrow edema (“Bone Marrow Edema profile”), joint fluid (“Effusion profile”) and malleolar groove shape (“Malleolar groove profile”) in each group compared to the other. The axis unit is the proportion of feature occurrence in relation to the total number of cases in each group, expressed as a decimal. Overlap indicates similarity. Coding of bone sections and joint fluid as described in Sect. 2.6

In Fig. 3, the group CI of three features (the posterior part of the lateral malleolus fibula [coded as Fibula B in Fig. 1], normal amount of synovial fluid [coded as Effusion 1] and concave malleolar groove) do not overlap, however after H-B without statistical significance. Distribution profiles for bone marrow edema, joint effusion and malleolar groove shape can be seen in the form of radar plots in Fig. 4. In the bone marrow edema profile, the control group is almost completely overlapping the split tear group, indicating a shared profile. The split tear profile, on the other hand, contains several areas of non-overlap (Fig. 4). The most notable are the posterior part of the lateral malleolus (coded as Fibula B, Fig. 1), lateral part of the talus (coded as Talus A, Fig. 1) and lateral part of the distal tibia (coded as Tibia A, Fig. 1) (Fig. 4).

**Fig. 5:**
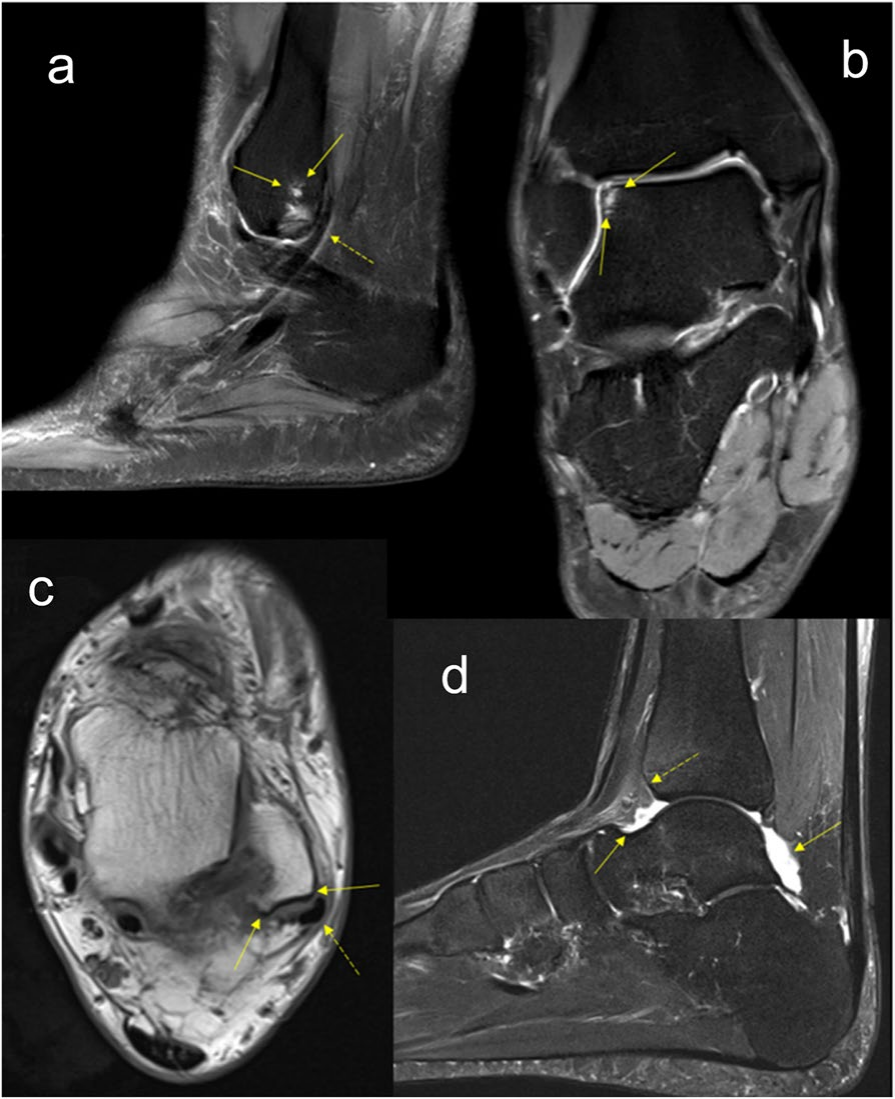
Collage of MR images exemplifying the features that were significantly more common in the split tear group. (a) Bone marrow edema in the posterior fibula. Note how the peroneus tendons pass directly behind the fibula (dashed arrow). (b) Bone marrow edema in the lateral talus. (c) Concave malleolar groove. Dashed arrow pointing at peroneus tendons. (d) Moderate joint effusion. Note the fluid expansion in both the anterior and posterior recess, but the anterior tibia is still in contact with fat tissue (dashed arrow) but not the talus (straight arrow). a – T2-weighted image with fat suppression, b – proton density-weighted image with fat suppression, c – T1-weighted image, d – T2-weighted image with fat suppression

The joint fluid profile illustrates how the control group leans more heavily towards no joint effusion (coded as Effusion 1) and the split tear group towards moderate joint effusion (Effusion 2) (Figs. 4 and 5).

The malleolar groove profile demonstrates how the malleolar groove shape was more often concave in the split tear group while the other two shapes were slightly more common in the control group, however no statistical significance was seen (Figs. 4 and 5; Table 3).

Significant differences in percentual feature occurrence between the two groups can be seen in Table 3. In the split tear group, bone marrow edema in the posterior fibula and lateral talus, moderate joint effusion and concave malleolar groove were more common (Fig. 5), however statistically significant differences after B-H were for bone marrow oedema in the posterior part of the lateral malleolus.

No joint effusion and history of trauma were significantly less common; 95% CI limits ranged from 2% to − 35% (Table 3). No other significant differences in percentual feature occurrence were found between the groups.

Several significant correlations between bone variables were found in both groups. The strongest correlations in the split tear group were a positive correlation (r = 0.4) between bone marrow edema in the medial part of the talus and normal joint fluid, a positive correlation (r = 0.32) between bone marrow edema in the anterior part of the lateral malleolus and no joint effusion, and a negative correlation (r = − 0.27) between bone marrow edema in the central part of the distal tibia and severe joint effusion.

In the control group, other correlations were predominant, with the strongest being a positive correlation (r = 0.27) between the presence of bone marrow edema in the medial talus and severe joint effusion. A negative correlation (r = − 0.22) was found between bone marrow edema in the medial talus and normal joint fluid. Correlations in the control group were weaker than those in the split tear group; none reached r ≥ 0.3. When the groups were combined, a negative correlation (r = − 0.15) was still found between bone marrow edema in the medial talus and no joint effusion. No correlation was found between bone marrow edema in the anterior fibula and joint effusion. The strongest correlation (0.31) was between medial talus and severe joint effusion. No other significant correlations were found.

## Discussion

We found significant differences between groups in the presence of bone marrow edema in the posterior part of the lateral malleolus, while there was no significant difference in occurrence of ATFL or CFL ruptures. Complete rupture of SPR was observed only in the control group. The most significant result of the current study is that bone marrow edema in the posterior half of the lateral malleolus is significantly common in patients with a peroneus split rupture. The absence of other significant differences between the groups confirms that the radiological diagnosis of split rupture is difficult.

We found several statistically significant correlations in the split tear group between the presence of bone marrow edema in the medial talus and a normal amount of synovial fluid.

Split of the PB is seen more often than that of PL, thus we focus on this pathology [3, 4]. There are certain skeletal MRI features related to PB split tears. Bone marrow edema in the posterior fibula probably corresponds to tendon vicinity [14]. The peroneus tendons pass directly behind the fibula while the lateral talus has no direct contact with the peroneus tendons but is located relatively close, hence the bone marrow edema could be related to split tears. The most common site of PB split rupture is located at the level of the tip of the apex of the lateral malleolus. A coincidence in occurrence with osteochondritis dissecans is possible since that lesion is usually also found in the talus [23]. It is interesting that moderate, but not severe, joint effusion was overrepresented in the split tear group. An increased amount of fluid in the joint cavity is associated with injury to the structures that limit or are in its lumen, like osteochondral injury [19]. Peroneus tendons run outside the joint cavity, on the articular capsule, thus probably the lack of a significantly increased amount of fluid in patients with peroneus split rupture. Because the amount of fluid is related to the degree of joint structure injury [19], the finding indicates a relationship between peroneus split tears and certain trauma types, but not severe trauma. Joint effusion is a relative common find however this variable was not statistically significant between groups after applying B-H. While trauma is considered one pathophysiological mechanism of a split rupture, significantly less history of trauma in the split tear group was somewhat expected as non-traumatic split tears are known. Most patients with split rupture do not memorize a precise ankle trauma, yet most of them report unspecific lateral ankle instability signs [24].

Beyond the significant differences, it is worth noting that most bone variables were similar in prevalence. This was generally expected, as most of the features had not been linked to peroneus split tears in previous research. Some features had been though (mainly os peroneum, prominent peroneal tubercle and lateral calcaneus bone marrow edema) [13] and finding no significant differences for these features was surprising.

The magnitude in differences between the groups can be interpreted by observing the CI. They ranged from 2% (lower limit, lateral talus) to − 35% (lower limit, no joint effusion) where the higher value would carry clinical relevance. Pinpointing the true difference based on our study is thus difficult but it does mean all differences have the potential to be substantial.

SPR disorder may cause peroneus tendon pathology inclusive of split rupture. SPR may be weakened or separated from the fibula following inversion ankle injuries. It may result as synovitis, subluxation or luxation of peroneus tendons, and split rupture [24]. Injuries of the lateral ligaments of the ankle are mainly related to acute trauma [2]. The most common mechanism of the ankle is inversion [25]. A about 3/4 of patients report residual symptoms up to 4 years after the injury [1; 3]. Split rupture of the PB may be responsible for some of the unspecific symptoms [24, 26]. However, low-grade ATFL, CFL or SPR injury symptoms may overlap with peroneus split rupture. Our groups differed in the incidence of SPR lesions, while injuries of ATFL or CFL did not differ statistically between the groups. This means that there is a relationship between PB split rupture and SPR lesions.

Most split ruptures of the PB are chronic, while ligament injuries are more associated with trauma [26, 27]. The history of ankle injury varied between our groups and was less common in the experimental group. Split rupture of the PB may result from the instability that results from ligament damage. Finally, anatomical connections between SPR and ligamentous structures have been investigated before, which may also contribute to the coexistence of SPR, ATFL and CFL injuries [28].

We found no differences in the anthropometric variables between the groups. However, it is difficult to determine based on our results whether BMI can contribute to split rupture of the PB. Studies in a more diverse cohort are needed to establish a relationship between BMI and the risk of split rupture of the PB.

PB split rupture is multifactorial and can be difficult to diagnose [26], so accompanying features are valuable. Ankle MRI is an appropriate imaging modality for the assessment of PB tendon pathology [26]. Several MRI-associated features of PB split tear were found in our study while the strongest positive correlation was noticed between medial talus bone marrow edema and a normal amount of synovial fluid. A negative correlation for the same features was found in the control group. This was the most distinct correlation in the PB split tear group compared to the control group. The pathophysiological relationship is unclear; however, it might suggest a multifactorial nature.

The distribution of peroneal groove shapes in neither group matched what was described by Edwards in 1928 (82% concave, 11% flat, 7% convex) [29] which was unexpected. Since they examined cases without any specific history of disease, we believe the control group is still appropriate for comparison. The results of their study relied on cadaveric studies of the entire fibula which in theory could yield a more comprehensive appreciation of the peroneal groove shape, as opposed to our MRI in vivo evaluation. Thereby, our results differ from the ruling theory that convex and flat grooves lead to peroneal split tears through overcrowding and mechanical stress [5]. We believe that peroneus split rupture is multifactorial and is related to static and dynamic factors. Although we included soft tissue and bone variables in the study, only the bone marrow edema in the posterior half of the lateral malleolus differentiated the groups after applying the B-H method.

From a pathophysiological perspective, one could speculate why a concave malleolar groove would predispose the peroneus tendons to tear. It is possible that some concave malleolar grooves contain sharp ridges, which in combination with repetitive subluxation or trauma could injure the peroneal tendons. Galli et al. [11] found correlations between peroneal split tear, undulating malleolar groove and osteophytes in the groove, thus alluding to osseus protrusions playing a role in the pathophysiology of peroneus tendon split tears. They did not, however, find any correlation with concave malleolar groove specifically.

Peroneus split tears most commonly do not cause acute symptoms [30]; it is likely that some of the cases were asymptomatic or that symptoms were unclear or diffuse. While all MRI scans were performed on clinical indication, not all indications were related to peroneus split tears. MRI examinations may have been conducted for any indication related to the ankle. The sample was still inherently comprised of cases referred on clinical indication, realistically entailing a higher proportion of symptomatic patients (e.g., ankle pain, instability). In contrast, if patients were recruited from the general population, the proportion of asymptomatic patients would likely be higher. Maybe more importantly, it is unknown whether asymptomatic and symptomatic peroneus split tears share the same pathophysiological mechanisms or form distinct groups.

Despite the limited number of subjects, the study still detected statistically significant finding and, relatively speaking, the sample size was substantial. The research to date has tended to only include case series (for example Sobel et al. n = 14 [4], Rademaker et al. n = 9 [18]) and has not dealt with statistical significance. Some studies have had larger sample sizes (for example Galli et al. n = 108 [11], Ersoz et al. n = 69 [17]) but have failed to include substantial numbers of peroneus split tears (n = 4 and n = 7, respectively). Bojanić et al. [8] had the largest number of peroneal split tears (n = 34), but their study used tendoscopy instead of MRI. We have not found any previous studies including a control group. Consequently, this study possibly includes the largest number of MRI cases with peroneus split tears to date.

There are some limitations to the study. During evaluation of the MR images, we noticed the shape of the malleolar groove changing noticeably in the longitudinal axis. Even when adhering to the defined level of where the PTFL attached, the shape could change from concave in the upper region to convex in the lower region. This would explain why Cohen’s kappa was the lowest for malleolar groove shape. Future studies could use a stricter definition of groove level or, alternatively, use 3D imaging for a more comprehensive view of the malleolar groove. The retrospective character, the lack of surgical correlation and the absence of a sample size calculation may also be limitations of this study.

Our study reaffirms that the ankle is one of the most challenging structures to evaluate on MRI. If some of the mentioned adjustments were made, we believe higher inter-rater reliability could be achieved.

Split rupture of PB is more common than that of PL [3, 4]. We included patients with PB split rupture; however, bone marrow edema may also be related to PL split rupture [14]. It is possible that split tears of the peroneus tendons share similar MRI features, but that conclusion cannot be drawn based on this study. Further investigations with a PL split rupture cohort are needed.

The presence of bone marrow edema in the posterior half of the lateral malleolus should be regarded as a potential “red flag” indicating the presence of PB split rupture. There is seemingly a lack of studies on the area, and we believe this study has laid some groundwork for future research.

## Conclusion

Bone marrow edema in the posterior fibula is significantly associated with peroneus split rupture. A distinct correlation was found between bone marrow edema in the medial talus and no joint effusion in patients with peroneus split rupture. These could potentially facilitate MRI assessment, enabling faster and more accurate diagnosis for patients, but prospective studies are required for causal corroboration. Beyond a shadow of a doubt, this study suggests that certain MRI features are associated with peroneus tendon split tears.

## Data Availability

The datasets used and/or analysed during the current study are available from the corresponding author on reasonable request.

## Abbreviations

ATFL: Anterior talofibular ligament
BMI: Body mass index
CFL: Calcaneofibular ligament
CI: Confidence intervals
ER: Emergency room
FOV: Field of view
IOC: International olympics committee
MRI: Magnetic resonance imaging
PACS: Picture archiving and communication system
PB: Peroneus brevis tendon
PD: Proton density
PL: Peroneus longus tendon
RIS: Radiology information system
SPR: Superior peroneal retinaculum
SPSS: Statistical package for the social sciences
SU: Sahlgrenska University Hospital
TE: Echo time
TR: Repetition time
TSE: Turbo spin echo
